# Relating resting-state hemodynamic changes to the variable language profiles in post-stroke aphasia

**DOI:** 10.1016/j.nicl.2018.08.022

**Published:** 2018-08-21

**Authors:** Ying Zhao, Matthew A. Lambon Ralph, Ajay D. Halai

**Affiliations:** a Neuroscience and Aphasia Research Unit, School of Biological Sciences, University of Manchester, UK;; b Department of Psychology, University of Cambridge, UK; c MRC Cognition and Brain Sciences Unit, University of Cambridge, UK

**Keywords:** Stroke aphasia, Lesion, Resting-state fMRI, Hemodynamic changes

## Abstract

Linking both structural lesions and the functional integrity of remaining brain tissue to patients' behavioural profile may be critical in discovering the limits of behavioural recovery post stroke. In the present study, we explored the relationship between temporal hemodynamic changes and language performance in chronic post-stroke aphasia. We collected detailed language and neuropsychological data for 66 patients with chronic (>1 year) post-stroke aphasia. We used principal component analysis to extract their core language-neuropsychological features. From resting-state fMRI scans in 35 patients, we calculated the lag in the time-course of the intact brain voxels in each patient. Finally, variation across the language-cognitive factors was related to both the patients' structural damage and the time-course changes in each patient's intact tissue. Phonological abilities were correlated with the structural integrity of the left superior temporal, angular gyrus, supramarginal gyrus and arcuate fasciculus regions and hemodynamic advance in the left intra-parietal sulcus. Speech fluency related to integrity of premotor regions, plus hemodynamic advance in the left middle/superior temporal gyrus, left middle occipital gyrus, and right angular gyrus. Semantic performance reflected a combination of medial ventral temporal lobe status and hemodynamic delay in the left posterior middle temporal gyrus. Finally, executive abilities correlated with hemodynamic delay in the left middle/inferior frontal gyrus, right rolandic operculum, bilateral supplementary motor areas/middle cingulum areas, and bilateral thalamus/caudate. Following stroke, patients' patterns of chronic language abilities reflects a combination of structural and functional integrity across a distributed network of brain regions. The correlation between hemodynamic changes and behaviours may have clinical importance.

## Highlights

• Examination of the functional integrity of remaining brain tissue in post stroke aphasia.
• Mapping lag in the time-course of the intact brain voxels from resting-state fMRI scans.
• Functional integrity was related to patients' language-cognitive abilities.
• Patients' language abilities reflect a combination of structural and functional integrity.

## 1 Introduction

Measuring changes in resting-state functional MRI (rs-fMRI) may provide insights into recovery mechanisms after brain damage (Carter et al., 2010; Carter et al., 2012; Grefkes and Fink, 2011, Grefkes and Fink, 2014; Ovadia-Caro et al., 2014). There are some advantages in collecting rs-fMRI data in patient populations as the technique can non-invasively measure hemodynamic responses of patients (Biswal et al., 1995) and the participant is simply asked to remain still in the scanner for around six minutes (Ovadia-Caro et al., 2014). These features suggest that the use of rs-fMRI might be one of the easiest and most feasible approaches to identifying functional networks in patient groups (Tie et al., 2014) and thus provide important information for possible recovery studies. Rs-fMRI studies have related changed functional connectivity (FC) to deficits and recovery mechanisms post-stroke, in motor deficits (Park et al., 2011) spatial neglect (He et al., 2007) and aphasia (Nair et al., 2015). There are, however, new ways to utilise rs-fMRI data, which might provide important insights about the relationship between patient's behavioural profile and the functional status of the remaining, intact brain regions.

Recently, rs-fMRI data were used to identify temporal hemodynamic changes in post-stroke populations (and proved crucial for understanding the true basis of altered FC) (Amemiya et al., 2014; Lv et al., 2013; Siegel et al., 2016). Intact brain regions had altered temporal hemodynamic flow, which either led or lagged behind the mean time course of intact grey matter. This finding was replicated in the acute and chronic stages of stroke, and steno-occlusive vessels disease (Amemiya et al., 2014; Siegel et al., 2016). Hemodynamic changes could emerge for at least two possible reasons. First, some brain regions share a vascular supply and therefore, if a common vascular supply is compromised, intact regions' hemodynamic response could be affected (Amemiya et al., 2014; Lv et al., 2013; Siegel et al., 2016). Secondly, remote regions can have structural and/or functional connections with the affected regions (Bullmore and Sporns, 2009) which could affect their functional integrity even if they have different vascular supplies. If temporal hemodynamic changes are caused by changes in blood flow to intact regions, it is interesting to explore which of these regions are related to behavioural performance.

There is a long history of relating patients' behavioural profiles to the underlying brain damage, both through classical lesion overlap mapping (Damasio et al., 1996) and more contemporary lesion-symptom analytic techniques (Bates et al., 2003). Whilst the location and extent of lesions are clearly a critical element in explaining patients' behavioural presentation, functional changes in the intact tissue may also be crucial. The core hypothesis of the current study is that patients' performance may reflect a combination of the direct effect of the lesion to the cognitive network-of-interest plus the functional status of the remaining cognitive networks. Accordingly, in this study we explored whether the structural lesion and hemodynamic lag in the intact regions (derived from rs-fMRI) could explain patient variation across four core components of language function. More specifically, there are two different kinds of change that might emerge after brain damage. First, it has become increasingly clear that many cognitive functions, including different aspects of language, are supported by distributed neural networks (e.g. Friederici and Gierhan, 2013). This network-based function may mean that when one or more of its nodes are damaged then the function of the entire network may become less efficient and time courses extended (Binney and Lambon Ralph, 2015) because there is reduced/inefficient activation propagation (Allendorfer et al., 2012; Geranmayeh et al., 2016; Skipper-Kallal et al., 2017). The second, additional possibility is that other cognitive functions (e.g. domain general executive control) become recruited in order to compensate or support the damaged system (Brownsett et al., 2013; Geranmayeh et al., 2014; Geranmayeh et al., 2017; Geranmayeh et al., 2016). To test the hypothesis, the current study determined the temporal hemodynamic changes in rs-fMRI data for a group of 35 patients with chronic post-stroke aphasia and related the results, alongside their structural lesions, to the pattern of their language and cognitive profiles.

## 2 Method

### 2.1 Participants

This study makes use of our large neuropsychological and neuroimaging dataset, consisting of 66 patients with chronic aphasia post-stroke (either ischaemic or haemorrhagic). The full database was used in the behavioural factor analysis. For the brain–behaviour correlational analyses, we utilised a subset of 35 patients who had both rs-fMRI as well as T1 scans (see Table 1 for a summary of demographic details). Recruitment criteria were: (1) monolingual native English speakers, (2) normal or corrected-to-normal hearing and vision, (3) right-handed, (4) one stroke, (5) at least 12 months' post-stroke and (6) no other known neurological conditions. All participants gave informed consent under approval from the local ethics committee. Structural imaging data from a healthy age and education matched control group (8 female, 11 male) was used to determine the lesion outline in the patients using an automated lesion identification toolbox (Seghier et al., 2008). We obtained control resting-state scans on a control group (N = 30), however they were not age-matched (mean age: 30.5 years, standard deviation: 5, range: 25–43 years). We did not use freely available resting-state data as we collected dual-echo fMRI data in order to improve signal loss in magnetic susceptible regions such the anterior temporal lobe (Halai et al., 2015; Halai et al., 2014).

**Table 1:** Participants' background information. Abbreviations (Boston Diagnostic Aphasia Examination: BDAE)

ID	Age	Gender	Education years	Post months	BDAE classification
1	44	M	11	40	Anomia
2	61	M	11	16	Broca
3	73	M	11	23	Mixed Non-fluent
4	53	F	11	47	Anomia
5	51	F	11	66	Anomia
6	54	M	13	35	Broca
7	77	F	11	56	Anomia
8	52	F	11	99	Mixed Non-fluent
9	69	F	19	39	Anomia
10	78	M	13	36	Mixed Non-fluent
11	68	M	11	21	Anomia
12	68	F	16	22	Anomia
13	59	M	13	37	Broca
14	59	M	11	34	Anomia
15	58	M	13	57	Global
16	51	M	13	72	Anomia
17	46	F	16	21	Conduction
18	82	M	10	13	Broca
19	79	M	11	64	Global
20	68	M	11	37	Conduction
21	44	F	13	37	Anomia
22	73	F	11	46	Transcortical motor aphasia
23	75	F	11	160	Mixed Non-fluent
24	84	M	9	35	Anomia
25	74	M	11	18	Global
26	43	F	16	15	Anomia
27	64	M	11	29	Mixed Non-fluent
28	67	M	11	44	Mixed Non-fluent
29	79	M	11	63	Mixed Non-fluent
30	45	M	11	25	Anomia
31	58	F	11	278	Anomia
32	67	M	11	13	Conduction
33	52	M	11	73	Global
34	86	M	9	17	Anomia
35	73	M	11	114	Broca

### 2.2 Neuropsychological assessments and analysis

The neuropsychological battery was designed to assess input/output phonological processing, semantic processing and sentence comprehension, and general cognitive function. The battery included tasks from the Psycholinguistic Assessments of Language Processing in Aphasia (PALPA) battery (Kay et al., 1992): (1) auditory discrimination using non-word minimal pairs, (2) auditory discrimination using word minimal pairs, (3) immediate repetition of non-words, (4) immediate repetition of words, (5) delayed repetition of non-words, and (6) delayed repetition of words. Tasks from the 64-item Cambridge Semantic Battery (Bozeat et al., 2000) included: (7) spoken word-to-picture matching, (8) written word-to-picture matching, (9) Camel and Cactus Test (picture), and (10) picture naming. Other language tasks included (11) the Boston Naming Test (BNT) (Kaplan et al., 1983) (12) written 96-trial synonym judgement (Jefferies et al., 2009) (13) the spoken sentence comprehension task from the Comprehensive Aphasia Test (CAT) (Swinburn et al., 2005) and the ‘Cookie theft’ picture description task from the Boston Diagnostic Aphasia Examination (Goodglass and Kaplan, 1983) Patients' responses from this picture description were recorded and transcribed. The (14) number of word tokens (T), (15) type/token ratio (TTR), (16) mean length of utterance in morphemes (MLU), and (17) words-per-minute (WPM) were computed. Cognitive assessments included (18) forward and (19) backward digit span (Wechsler, 1987) (20) the Brixton Spatial Rule Anticipation Task (Burgess and Shallice, 1997) and (21) Raven's Coloured Progressive Matrices (Raven, 1962) All scores were converted into percentage based on the maximum score available; where no maximum was available, we used the max score in the group.

Following our previous studies (Butler et al., 2014; Halai et al., 2017), we used principal component analysis (PCA) with varimax rotation (SPSS 22) on all 66 patients' scores to obtain robust and interpretable core language-cognitive components. Factors with eigenvalues >1.0 were rotated with varimax rotation. Individual patient factor scores were obtained (regression method) allowing us to place each case along a continuum of scores for each factor (Table 3). Each behavioural factor is statistically independent (orthogonal) and so, when correlated against the lesion or hemodynamic data, the identified brain regions represent areas that are uniquely related to each underlying behavioural factor.

### 2.3 Acquisition of neuroimaging data

High resolution structural T1-weighted Magnetic Resonance Imaging (MRI) scans were acquired on a 3.0 Tesla Philips Achieva scanner (Philips Healthcare, Best, The Netherlands) using an 8-element SENSE head coil. A T1-weighted inversion recovery sequence with 3D acquisition was employed, with the following parameters: TR (repetition time) = 9.0 ms, TE (echo time) = 3.93 ms, flip angle = 8°, 150 contiguous slices, slice thickness = 1 mm, acquired voxel size 1.0 × 1.0 × 1.0 mm^3^, matrix size 256 × 256, FOV = 256 mm, TI (inversion time) = 1150 ms, SENSE acceleration factor 2.5, total scan acquisition time = 575 s.

The rs-fMRI were obtained with a dual-echo EPI technique to improve signal detection within inferior temporal and orbitofrontal regions (Halai et al., 2015; Halai et al., 2014; Poser et al., 2006) We used the following parameters: TEs = 12 and 35 ms, TR = 2.8 s, voxel size = 3 × 3 × 4 mm, FOV = 240 × 124 × 240 mm, matrix size = 80 × 80, flip angle = 85°. A total of 130 volumes were collected over 6.25 min. The FOV was tilted (up to) 35° off the AC-PC to minimise ghosting artefacts on the temporal lobes (Halai et al., 2014) Participants were instructed to look at the fixation cross and lie still during scanning.

### 2.4 Preprocessing neuroimaging data

High-resolution structural scans were pre-processed with the same procedure as our previous studies (Butler et al., 2014; Halai et al., 2017) using Statistical Parametric Mapping software (SPM8: http://www.fil.ion.ucl.ac.uk/spm/) and a modified segmentation-normalisation program (Seghier et al., 2008). After normalising individual lesioned brain images into standard Montreal Neurological Institute (MNI) space, images were smoothed with an 8 mm full-width-half-maximum (FWHM) Gaussian kernel. Lesions were automatically identified for each patient by comparing the structural image with an age and education matched control group, using an outlier detection algorithm to identified ‘abnormal’ voxels (Seghier et al., 2008). All parameters were kept at default except the lesion definition ‘U-threshold’, which was modified from 0.3 to 0.5 after comparing the results obtained from a sample of patients to what would be nominated as lesioned tissue by an expert neurologist. The overall lesion maps are shown in Fig. 2A.

Resting-state analysis was performed using SPM8 and the Data Processing Assistant for Resting State fMRI (DPARSF Advanced Edition, version 2.3) toolbox (Yan and Zang, 2010). Pre-processing of the functional data included discarding the first two time-points, slice time correction, volume realignment, dual-echo linear average combination, and co-registration to the T1. All functional data were inspected using an artefact detection tool (ART; http://www.nitrc.org/projects/artifact_detect/) to identify time-points with high motion and/or signal artefacts. Outlier time-points were defined as ±2.5 mean intensity (*z*-score) and >1 mm movement in any direction (outliers were saved as separate regressors). In addition to regressing outlier time-points, we added 24 movement parameters as regressors in order to account for movement related artefacts (Ashburner and Friston, 1999). We then used DPARSFA to regress the influence of nuisance variables (mean CSF signal, 24 movement parameters, outlier signal intensity volumes and outlier movement volumes). We did not include mean white matter and global signal in this regression in accordance with the preprocessing requirements to calculate hemodynamic changes (Siegel et al., 2016). Finally, images were normalized by using the T1 image unified segmentation transformation matrix, obtained from Seghier and colleagues' algorithm (Seghier et al., 2008), smoothed using 8 mm FWHM Gaussian kernel and temporally filtered between 0.01–0.08 Hz.

### 2.5 Hemodynamic changes calculation

**Fig. 1:**
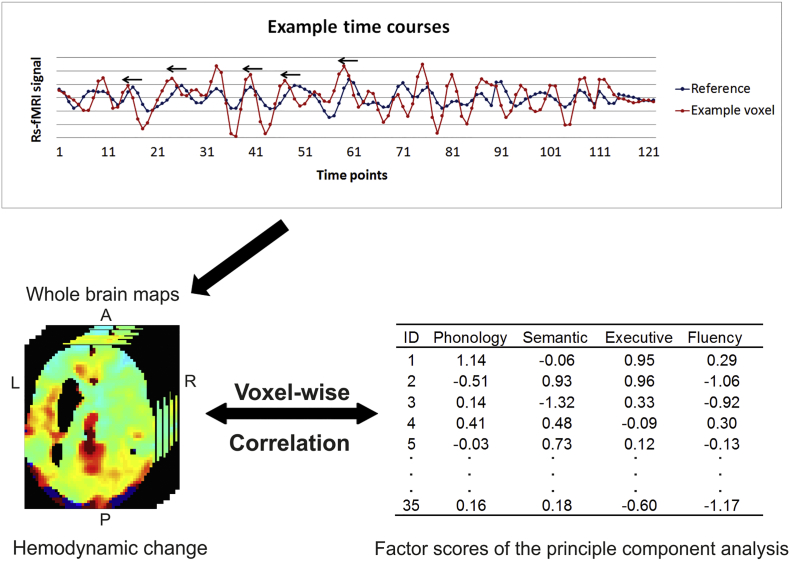
Main analysis. The top panel is an example time course of a voxel and the reference signal. The example voxel appears to be shifted to the left, leading before the reference signal. The bottom panel is the correlational analysis between voxel-wise homonymic changes and factor scores across patients who had intact tissue for each voxel.

The preprocessed resting-state data were resliced to 48 × 64 × 48 in order to match the dimensions in the Lag-suite program (Siegel et al., 2016). The analysis firstly calculates the mean time course of intact grey matter regions across the whole brain as the reference signal (see Fig. 1 for illustration). This is a suitable reference because it is closest to the null hypothesis that there are no differences in hemodynamic responses across intact voxels (Siegel et al., 2016). Previous studies have used a time window of around 8 s (Bonakdarpour et al., 2007; Siegel et al., 2016); therefore we have used a time window of 8.4 s (± 3 TRs). The program then determines which time shift enables the maximum cross-correlation C_i_(τ) using a sliding window of 1 TR based on the formula (Mitra et al., 2014):

$$C_{i}\left(\tau \right)=\left(1/n_{\tau }\right)\sum_{t}\left[\frac{g\left(t\right)∙s_{i}\left(t+\tau \right)}{\sigma_{g}\;\sigma_{s_{i}}}\right]$$

where g is the reference signal, s_i_ is the signal in voxel i, and σ_g_ and σ_s_i__ are the standard deviations of the two signals. t is the remaining time-points and n_τ_ is the number of frames included after a shift of τ (−8.4 s to 8.4 s). Thirdly, the Lag-suite fits various values of C_i_(τ) into a parabolic function, so that the C_i_(τ^m^) can be modelled and the corresponding temporal shifts τ^m^ computed. The temporal shift τ^m^ is considered as the temporal hemodynamic change for voxel i. Directionality is important, where positive and negative τ^m^ corresponds to a lag or lead relative to the mean grey matter signal, respectively. Voxels within the lesion were excluded from the correlation analysis. Furthermore, voxels that were not positively correlated with the reference signal (r < 0.1) between the intervals, −8.4 s to 8.4 s were also excluded as these voxels do not show hemodynamic change using the current reference signal. The temporal hemodynamic changes were calculated for all intact grey matter voxels.

### 2.6 Voxel-wise hemodynamic change, voxel integrity (lesion) and behaviour mapping

The relationship between patients' structural lesions and the language-cognitive factors was established in the same way as our previous studies (Butler et al., 2014; Halai et al., 2017). We used the T1-weighted images with continuous signal values across the whole brain and correlated these with the behavioural factor scores using the voxel-based correlational methodology (VBCM) (Tyler et al., 2005), a variant of voxel-lesion symptom mapping (VSLM) (Bates et al., 2003). In order to control for lesion size, each participants' lesion volume was calculated from the automated lesion identification method (Seghier et al., 2008) and this was entered as a covariate in each VBCM. The resultant factor-lesion maps (Fig. 3) were thresholded at AlphaSim cluster corrected p < 0.01 with voxel p < 0.005. AlphaSim correction is a cluster thresholding method developed in software Analysis of Functional NeuroImages (AFNI) which uses a Monte Carlo method to compute cluster-size probability values (Cox, 1996).

**Fig. 2:**
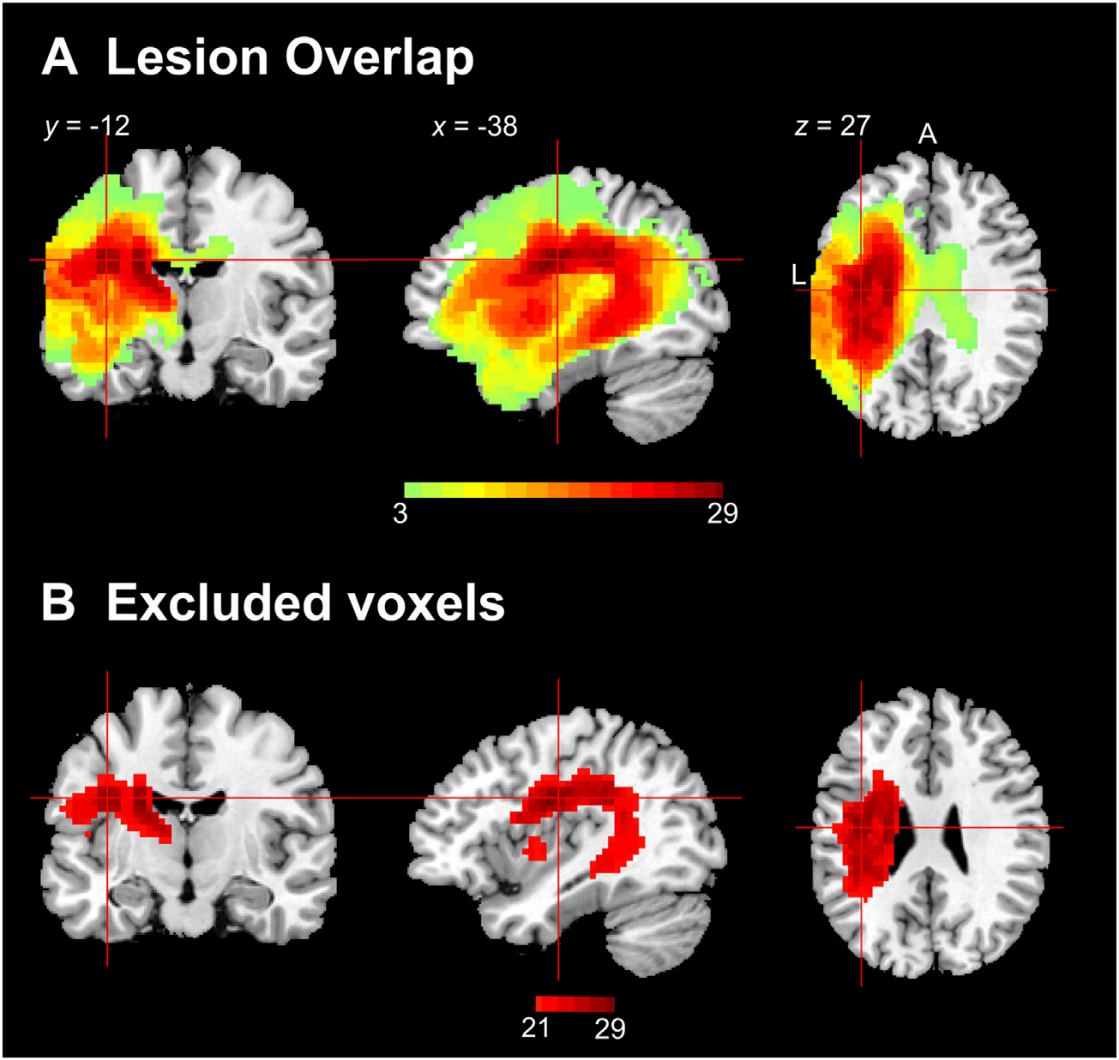
(A) Lesion distribution of 35 patients. (B) Excluded voxels which were damaged in >20 patients.

We conducted a Pearson correlation analysis to determine which voxel hemodynamic changes were related to behavioural performance. To control for implausible hemodynamic changes, we excluded voxels whose lag/lead values were beyond ±3 standard deviations. Similarly, we removed patients' who had a factor score beyond ±3 standard deviations, reducing the impact of extreme cases (only two were identified). Finally, in the brain and behaviour mapping stage, we conducted voxel-specific analyses whereby patients were only included if they had intact tissue in that voxel (avoiding the situation were a lesioned voxel is included in the correlation analysis). To avoid small samples, the correlational analysis was conducted on voxels that were intact in at least 15 patients. Accordingly, if >20 patients had damage in a given voxel, that voxel was excluded from the correlational analysis (Fig. 2B. shows excluded voxels). The sample 15 size was estimated based on: power = 0.8, correlation = 0.75, and significance level = 0.01. The correlational analysis threshold was set at AlphaSim cluster-corrected p < 0.01 with voxel p < 0.01. We also repeated the analysis using FDR correction (FDR corrected voxel-wise p < 0.05 and cluster >50 voxels).

## 3 Results

### 3.1 Lesion distribution and exclusion mask

The lesion overlap of the 35 patients is shown in Fig. 2A. Excluded voxels, damaged in >20 patients are shown in Fig. 2B. Excluded regions included a part of the insular, rolandic operculum, caudate, thalamus, postcentral gyrus, and putamen – representing the regions that are most commonly affected after MCA stroke (Phan et al., 2005).

### 3.2 Behavioural performance

PCA revealed four orthogonal dimensions (see Table 2) and replicates findings from our previous studies (Halai et al., 2017). The first factor loaded highest with repetition but also with naming and digit span (and weakly with spoken sentence comprehension) suggesting this factor relates to phonological ability. The second factor loaded highest with picture matching but also with synonym judgement, the Camel and Cactus Test and type/token ratio implying this factor relates to semantic processing. The third factor loaded highest with minimal pairs, Ravens coloured progressive matrices and the Brixton spatial anticipation test suggesting that this factor relates to executive-related problem-solving or decision-making processes. Finally, the fourth factor loaded highest with the number of speech tokens produced but also with words per minute and mean length of utterances, which suggests that this factor relates to the quantity of speech produced. Individual patient factor scores are shown in Table 3.

**Table 2:** Loadings of behavioural assessments on factors extracted from the varimax rotated PCA.

Tasks	Component			
	Phonology	Semantics	Executive	Fluency
Immediate Repetition - Non-words	**0.872**	0.073	0.242	0.154
Delayed Repetition - Non-words	**0.885**	0.042	0.242	0.154
Immediate Repetition - Words	**0.837**	0.225	0.136	0.196
Delayed Repetition - Words	**0.874**	0.238	0.189	0.213
64-Item Naming	**0.792**	0.454	0.156	0.135
Boston Naming Test	**0.816**	0.402	0.065	0.116
CAT Spoken Sentence Comprehension	**0.523**	0.467	0.427	0.133
Forward Digit Span	**0.765**	0.233	0.170	0.085
Backward Digit Span	**0.636**	0.161	0.130	0.302
Spoken Word to Picture Matching	0.236	**0.809**	0.252	0.122
Written Word to Picture Matching	0.178	**0.721**	0.497	0.147
96 Synonym Judgement	0.394	**0.677**	0.290	0.323
Camel and Cactus Test: Pictures	0.079	**0.700**	0.480	0.274
Type/Token ratio	0.358	**0.729**	−0.067	−0.083
Minimal Pairs - Non-words	0.384	0.072	**0.803**	−0.072
Minimal Pairs - Words	0.445	0.180	**0.693**	0.077
Raven's Coloured Progressive Matrices	0.035	0.268	**0.740**	0.179
Brixton Spatial Anticipation Test	0.116	0.176	**0.701**	0.240
Words-Per-Minute	0.353	0.103	0.078	**0.755**
Token	0.059	0.030	0.193	**0.876**
Mean Length of Utterance in Morphemes	0.360	0.268	0.111	**0.799**

Factor loadings >0.5 are given in bold; CAT = Comprehensive Aphasia Test.

**Table 3:** Participants' factor scores.

ID	F1 Phonology	F2 Semantics	F3 Executive	F4 Fluency
1	1.14	−0.06	0.95	0.29
2	−0.51	0.93	0.96	−1.06
3	0.14	−1.32	0.33	−0.92
4	0.41	0.48	−0.09	0.30
5	−0.03	0.73	0.12	−0.13
6	−1.81	−0.09	1.23	2.40
7	0.20	0.59	−0.16	−0.98
8	−0.77	1.27	−0.39	−1.14
9	−0.25	−0.01	0.32	***3.91***
10	−0.43	0.39	0.50	−1.54
11	1.15	−0.06	0.30	0.42
12	1.26	0.25	0.43	1.14
13	−0.87	0.97	0.24	−0.39
14	0.07	0.09	1.26	0.62
15	−1.56	−1.25	1.86	−0.77
16	−0.43	−0.04	0.66	1.42
17	−1.45	0.95	1.03	−0.35
18	0.12	0.94	0.18	−0.86
19	−0.09	***−3.88***	−0.54	−0.83
20	−1.07	0.56	0.25	0.29
21	0.44	0.33	1.14	−0.71
22	0.53	0.50	0.23	−1.02
23	−1.38	0.42	−0.42	−0.25
24	−0.27	1.62	−1.09	0.45
25	−0.69	−1.18	−1.31	−0.49
26	1.46	0.31	0.60	−0.47
27	−0.09	−2.31	1.34	−0.23
28	−0.65	−0.69	−0.13	−1.73
29	−0.23	−0.36	−2.27	−0.53
30	0.59	0.66	0.92	−0.12
31	0.86	0.81	0.01	−1.02
32	−1.58	0.25	1.10	0.80
33	−1.15	−2.45	1.29	−0.88
34	−0.03	0.43	−0.79	1.86
35	0.16	0.18	−0.60	−1.17
				

Scores beyond ±3 standard deviation from the mean are shown in bold italic

### 3.3 Voxel-wise hemodynamic change, structural lesions and behaviour mapping

**Fig. 3:**
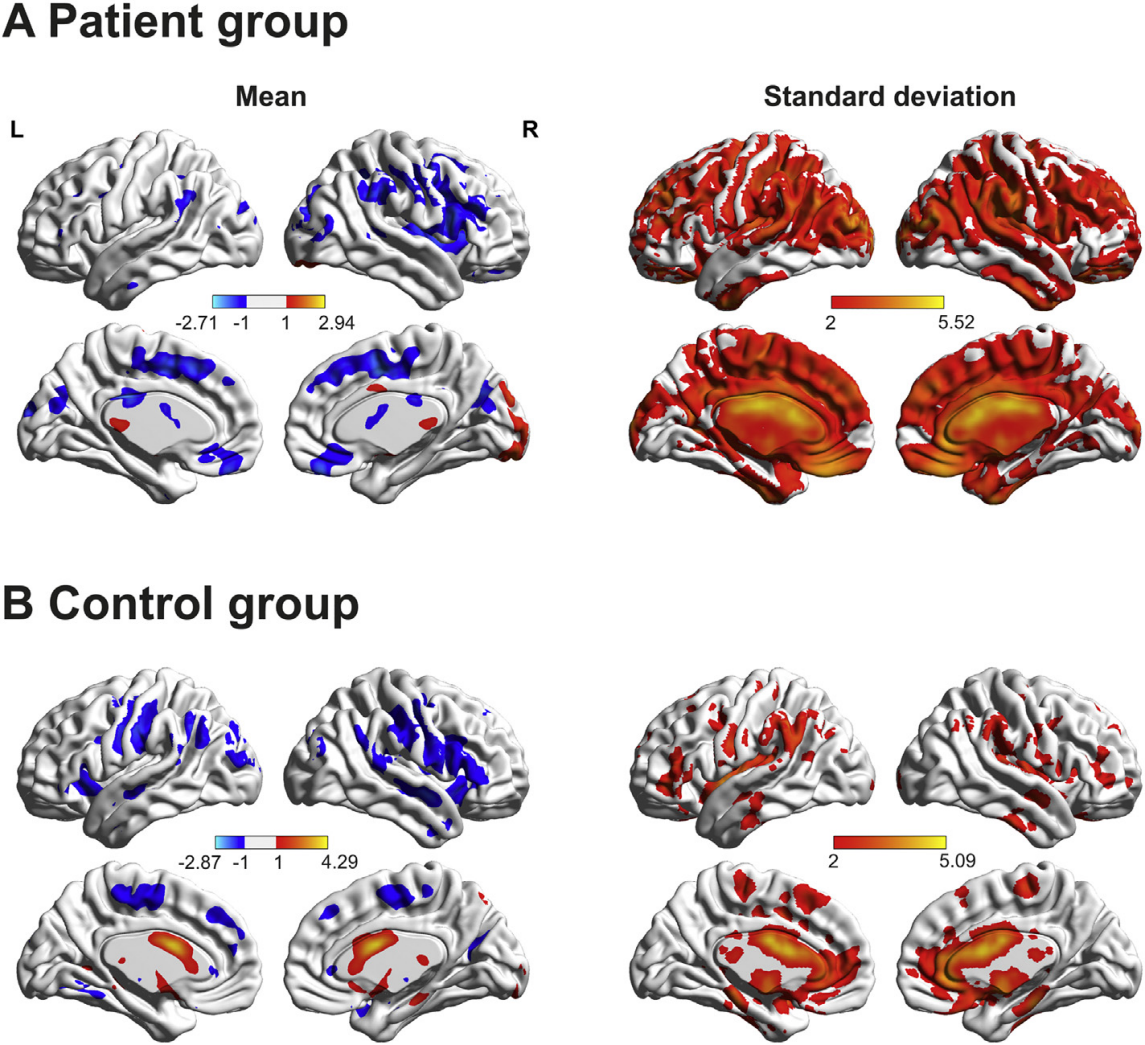
The mean and standard deviation hemodynamic lag for chronic post-stroke patients and control group. The Figure shows that the patient group have fewer hemodynamic changes in the left hemisphere (in the lesioned area), while having much more variability across the whole brain compared to controls.

To begin with, we determined the mean and SD of the hemodynamic lag for each voxel in the patient and control groups (see Fig. 3). For each patient, we only included non-lesioned voxels in the analyses. Fig. 3 shows that overall, the mean hemodynamic changes are similar across the two groups, except a noticeable difference in the left hemisphere where patients have damage to the MCA territory. In contrast, the SD for patients is much larger than for controls and this extends across the whole brain including areas outside of the lesion territory. This indicated that the hemodynamic profile had changed in the patients post stroke.

**Fig. 4:**
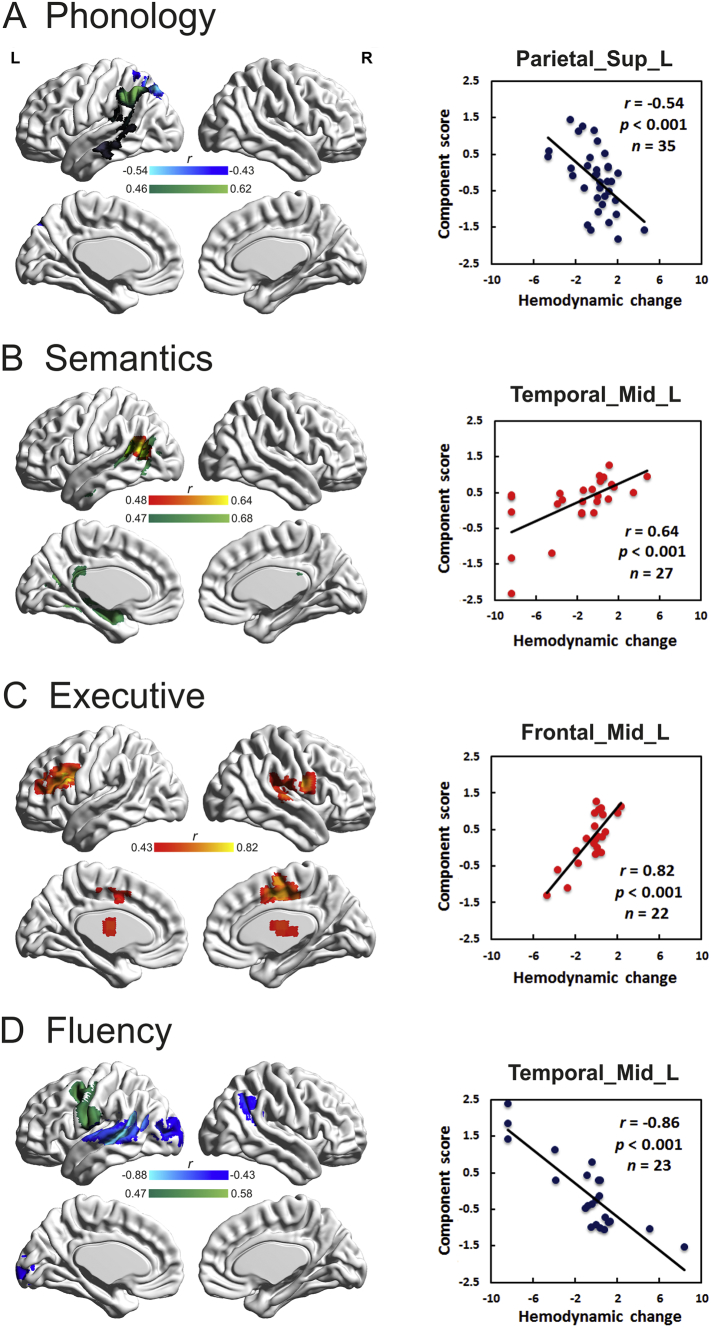
The left column shows voxel-wise correlations between hemodynamic changes/ grey matter intensity and factor scores. For correlations with hemodynamic changes, positive correlations indicating delay are shown in red; while negative correlations indicating advance are shown in blue (Alphasim cluster corrected p < 0.01 with voxel p < 0.01). Results of correlational analyses between grey matter intensity and factor scores are shown in green (Alphasim cluster corrected p < 0.01 with voxel p < .005). For phonology, dark grey indicates clusters that survived at a lower the threshold (Alphasim cluster corrected p < 0.01 with voxel p < 0.01). The right column shows the scatter plots of the peak voxel as an example to help with interpretation. (For interpretation of the references to colour in this figure legend, the reader is referred to the web version of this article.)

Fig. 4 shows the correlation analyses (A) between voxel damage and each factor score (green overlay) and (B) between hemodynamic changes and each factor score (hot and cold overlay), with a scatterplot of the peak voxel to aid interpretation. The direction of the correlation is important: a positive correlation suggests that the longer the lag within the area, the better the performance, whereas a negative correlation suggests that if the area is engaged earlier, performance improves.

The patients' phonological abilities were uniquely related to damage in the left angular gyrus, the supramarginal gyrus and the arcuate fasciculus (cluster size = 1128 voxels, 9024 mm^3^; peak coordinate: −50, −54, 48; r = 0.62, p < 0.001, N = 35), which also extended to the middle/superior temporal lobe at a lower threshold (AlphaSim cluster-corrected p < 0.01 and voxel p < 0.01). The phonology factor was negatively correlated with hemodynamic changes in the left superior/inferior parietal sulcus (cluster size = 75 voxels, 2025 mm^3^; peak coordinate: −29, −72, 52; r = −0.54, p < .001, N = 35).

The patients' semantic abilities were related to damage along the left middle temporal gyrus, medial temporal lobe and inferior longitudinal fasciculus to the anterior temporal lobe (cluster size = 4130 voxels, 33,040 mm^3^; peak coordinate: −30, −64, 22; r = 0.68, p < 0.001, N = 34). The semantic factor was positively correlated with hemodynamic changes in the left posterior middle temporal gyrus (cluster size = 61voxels, 1647 mm^3^; peak coordinate: −53, −57, 18; r = 0.64, p < 0.001, N = 27).

The executive factor did not correlate with the lesion profile; however, it positively correlated with hemodynamic changes in four clusters, including the left middle/inferior frontal gyrus (cluster size = 151, 4407 mm^3^; peak coordinate: −35, 36, 18; r = 0.82, p < 0.001, N = 22), the right rolandic operculum (cluster size = 159, 4293 mm^3^; peak coordinate: 55, −3, 21; r = 0.68, p < 0.001, N = 35), bilateral supplementary motor areas/middle cingulum areas (cluster size = 116, 3132 mm^3^; peak coordinate: 11, −3, 48; r = 0.70, p < 0.001, N = 34), and bilateral thalamus/caudate (cluster size = 82, 2214 mm^3^; peak coordinate: −14, −3, 24; r = 0.58, p < 0.001, N = 35).

The patients' fluency abilities were related to damage in middle frontal gyrus and precentral/postcentral gyrus (cluster size = 1468 voxels, 11,744 mm^3^; peak coordinate: −42, 4, 48; r = 0.58, p < 0.001, N = 34). The fluency factor was negatively correlated with the left middle/superior temporal gyrus (cluster size = 257; peak coordinate: −53, −39, 3; r = −0.86, p < 0.001, N = 23), the left middle occipital gyrus (cluster size = 93; peak coordinate: −41, −78, 6; r = −0.59, p < 0.0001, N = 31), and the right angular gyrus (cluster size = 67; peak coordinate: 46, −54, 36; r = −0.58, p < 0.001, N = 34). The results were very similar if, instead of AlphaSim correction, we applied FDR voxel-wise correction p < 0.05 with cluster >50.

## 4 Discussion

In order to gain a better understanding of recovery after brain injury, we need to move towards a model that incorporates functional changes following damage. Recent studies have shown that functional connectivity changes in resting-state fMRI can be attributed to deficits and recovery in motor, visual neglect and language abilities (He et al., 2007; Nair et al., 2015; Park et al., 2011). It has been shown that changes to functional connectivity may reflect altered hemodynamic responses (i.e., whether the regional responses are engaged faster or slower than normal: (Siegel et al., 2016). Accordingly, mapping these changes in hemodynamic responses could improve our understanding of the neural bases of post-stroke aphasia. In the current study we showed that the timing of the hemodynamic response function (which can either lead or lag behind the global mean) related to behavioural performance in regions outside of the core lesion correlates (identified through standard lesion-symptom mapping methods). For example, we found that phonological processing was related not only to the structural integrity of the inferior parietal region and arcuate fasciculus, but also to early hemodynamic engagement of the intra-parietal sulcus (IPS). By identifying both of these correlates simultaneously, we can start to build a more sophisticated picture of aphasia in which the location of the lesion and physiological changes in parts of the remaining tissue together inform our understanding of patients' performance.

In previously studies, the IPS has been found to be active in multiple domains, engaged in top down attention, executive semantics and phonological decisions (Humphreys and Lambon Ralph, 2014). During the recovery of post-stroke aphasia, the FC of this region with frontal regions was found to be increased when performing language tasks, as measured by task-state fMRI (Sharp et al., 2010). Our results suggest that faster blood flow in IPS or early involvement may allow for additional attention-executive processes to assist phonological processing.

The fluency factor was related to lesions in middle frontal gyrus and precentral/postcentral gyrus, plus earlier engagement of the left superior temporal gyrus, left occipital region, and right angular gyrus resulted in increased speech output. Previous research has associated the left auditory and vision sensory regions with monitoring sensory consequences of articulatory movements (e.g. seeing a speaker's face whilst listening) (Chesters et al., 2015; Jacks and Haley, 2015). A classic and well-known phenomenon is that delayed auditory and/or visual feedback disrupts fluent speech production in healthy participants, indicating that sensory regions contribute to successful speech output through online monitoring (Chesters et al., 2015). Our patient results are in line with these reports and suggest that if sensory regions have faster cerebral blood flow or are engaged earlier, there is sufficient auditory feedback to aid speech production.

Semantic ability was related to damage along the temporal lobe extending to the anterior temporal lobe. Beyond the lesion correlates, we identified that greater lag within the posterior middle temporal gyrus resulted in better semantic ability. The posterior middle temporal gyrus has been implicated in semantic control processes, which is a part of the wider semantic network (Jefferies et al., 2008; Lambon Ralph et al., 2016; Whitney et al., 2010). We hypothesise these regions may have slower blood flow or that the longer the region is engaged, the greater the influence of semantic control processes being applied to enable the correct shaping of semantic representations.

The lesion correlation analysis found no significant clusters with executive skill. There was, however, a correlation with hemodynamic changes. Here, greater hemodynamic lag within bilateral prefrontal, medial frontal, and thalamus regions related to better executive performance. This is consistent with a wide range of studies on healthy and patient groups, showing that these regions play a role in executive function (Botvinick et al., 2001; Caeyenberghs et al., 2014; Duncan and Owen, 2000; Kubat-Silman et al., 2002; Ridderinkhof et al., 2004). In the current study, we hypothesise that slower cerebral blood flow or longer engagement of these cognitive control regions (and a thus lagging time-course) leads to better executive ability.

One intriguing aspect of the results is the different correlation direction (negative or positive) across regions. We speculate that the direction of the correlation could relate to whether the region is part of the primary network or part of a supporting, secondary network (see Introduction). To clarify, regions outside the primary phonological (domain-general attention) and fluency (auditory feedback loop) network were associated with faster cerebral blood flow or engaged earlier (negative correlations), which led to better behavioural performance. Conversely, regions within the primary semantic (semantic control) or executive (multi-demand) network were associated with slower cerebral blood flow or engaged for longer (positive correlations), which led to better behavioural performance. The results suggest that, within a core functional network (e.g., semantics), damage to one or more of its nodes may make the remaining network relatively slow because activation cannot propagate around the network so efficiently. Interestingly, related results and proposed mechanism have been shown through combination of repetitive transcranial magnetic stimulation and fMRI in healthy participants (Binney and Lambon Ralph, 2015; Hallam et al., 2016; Jung and Lambon Ralph, 2016). In order to support good performance, the damaged network has to run for longer before the same output can be computed, and thus the hemodynamic response may be delayed. The pattern may be different when there is recruitment of another cognitive process in order to boost or support the damage function. Specifically, when the domain-specific network (e.g., for phonology) is undamaged then other cognitive domains (e.g., domain-general executive control) are not required. When the core network is compromised, however, these additional components may be upregulated in order to boost or correct the damaged system (Brownsett et al., 2013; Geranmayeh et al., 2014; Geranmayeh et al., 2017; Geranmayeh et al., 2016). This increased involvement may generate the speeded hemodynamic response observed in this study, especially if early engagement of the compensatory mechanisms generates better performance. Further research is required to verify this hypothesis. There are, of course, obvious implications for potential targets for therapy and intervention – in which non-invasive brain stimulation methods, e.g., transcranial magnetic stimulation and transcranial direct-current stimulation (Chieffo et al., 2013; Fridriksson et al., 2011) might be used to modulate the hemodynamic lag of these critical regions.

Finally, beyond rs-fMRI, temporal hemodynamic changes have also been reported in task-based fMRI studies of post-stroke patients. The hemodynamic response function (HRF) of some stroke aphasia patients has been shown to be delayed in its time to peak and had a reduced amplitude (Bonakdarpour et al., 2007). A second study used fMRI to scan patients with left hemisphere stroke while overtly producing words before and after therapy (Peck et al., 2004). The HRF time to peak in right hemisphere regions, e.g., Broca's area homologue and supplementary motor area, was found to have decreased after therapy (Peck et al., 2004). The hemodynamic changes in resting-state and task-state could have a common cause. As noted in the Introduction, a common vascular supply could be damaged to affect intact regions, or intact regions' HRF may be disrupted when there is to damage to a functionally or structurally connected area. Although we do not know exactly how the changes in hemodynamic responses reflect the underlying neuronal activity, we note that detecting hemodynamic changes may provide a target for future directions in aphasia treatment (see above). It should be noted that not all patients show hemodynamic changes in task-state fMRI or resting-state fMRI (Bonakdarpour et al., 2007; Siegel et al., 2016). Therefore, the temporal hemodynamic change may be one additional, separate factor related to aphasia profile.

## 5 Limitations

There are some limitations in the current study. First, we do not have any direct measurements of the status of the arteries using magnetic resonance angiography (MRA) images or similar methods, and therefore cannot rule out arterial stenosis. Future studies may consider its influence on hemodynamic changes. Second, the time window that we used to estimate lag is based on a TR of 2.8 s, which is somewhat longer than the 2 s used in previous studies (Siegel et al., 2016). Our TR was constrained by our fMRI protocol in which we collected dual-echo data allowing us to image magnetic susceptible regions better (Halai et al., 2015; Halai et al., 2014). As technologies improve, e.g., multi-banding multi-echo fMRI, the TR can be reduced much further while maintaining good signal coverage (see Kundu et al. (2017) for a review).

## 6 Conclusion

In conclusion, our study established the relation between behaviour valances and hemodynamic changes of intact regions post stroke. It can help us to understand a potential mechanism for compensation and intervention.
